# Correction: Cytochrome P450 metabolic resistance (CYP6P9a) to pyrethroids imposes a fitness cost in the major African malaria vector *Anopheles funestus*

**DOI:** 10.1038/s41437-020-00360-2

**Published:** 2020-09-02

**Authors:** Magellan Tchouakui, Jacob Riveron Miranda, Leon M. J. Mugenzi, Doumani Djonabaye, Murielle J. Wondji, Micareme Tchoupo, Williams Tchapga, Flobert Njiokou, Charles S. Wondji

**Affiliations:** 1 Centre for Research in Infectious Diseases (CRID), P.O. Box 13501, Yaoundé, Cameroon; 2 Parasitology and Ecology Laboratory, Department of Animal Biology and Physiology, Faculty of Science, University of Yaoundé 1, P.O. Box 812, Yaoundé, Cameroon; 3 Department of Vector Biology, Liverpool School of Tropical Medicine, Pembroke Place, L35QA Liverpool, UK; 4 Department of Biochemistry and Molecular Biology, Faculty of Science, University of Buea, P.O. Box 63, Buea, Cameroon; 5 Pharmacology and Toxicology Laboratory, Department of Biochemistry, Faculty of Science, University of Yaoundé 1, P.O. Box 812, Yaoundé, Cameroon

Correction to: *Heredity*

10.1038/s41437-020-0304-1

This article was originally published Online First without Open Access. After publication in volume 124, issue 5, page 621–632 the author decided to opt for Open Choice and to make the article an Open Access publication. Therefore, the copyright of the article has been changed to © The Author(s) 2020 and the article is forthwith distributed under the terms of the Creative Commons Attribution 4.0 International License, which permits use, sharing, adaptation, distribution and reproduction in any medium or format, as long as you give appropriate credit to the original author(s) and the source, provide a link to the Creative Commons license, and indicate if changes were made. The images or other third party material in this article are included in the article’s Creative Commons license, unless indicated otherwise in a credit line to the material. If material is not included in the article’s Creative Commons license and your intended use is not permitted by statutory regulation or exceeds the permitted use, you will need to obtain permission directly from the copyright holder. To view a copy of this license, visit http://creativecommons.org/licenses/by/4.0/.

